# Control of Tire Wear Particulate Matter through Tire Tread Prescription

**DOI:** 10.3390/polym15132795

**Published:** 2023-06-23

**Authors:** Jin U. Ha, Seok H. Bae, Yu J. Choi, Pyoung-Chan Lee, Sun K. Jeoung, Sanghoon Song, Choong Choi, Jae S. Lee, Jaeyun Kim, In S. Han

**Affiliations:** 1 Chassis & Materials Research Laboratory, Korea Automotive Technology Institute, Cheonan-si 31214, Republic of Korea; 2 School of Chemical Engineering, Pusan National University, Busan 46241, Republic of Korea; 3 R&D Department, Hankook Tire & Technology, Daejeon 34127, Republic of Korea; 4 R&D Department, OCI, Sungnam-si 13212, Republic of Korea; 5 R&D Department, Kumho Petrochemical, Daejeon 34044, Republic of Korea; 6 Interior & Exterior Materials Development Team, Hwaseong-si 18280, Republic of Korea

**Keywords:** particulate matter, tire, wear, abrasion, wear simulator, truck bus radial

## Abstract

This study aims to analyze tire wear particulate matter (TWP) from tread rubber with different formulations and to compare the concentration of TWP with different wear devices. The TWP generated during the abrasion of truck and bus radial (TBR) tires were examined, and the effect of using different types of rubber and carbon black (CB) were investigated. When natural rubber (NR) was solely used as the tire tread rubber material, there was a higher concentration of 5–10 µm TWP. However, when the tread formulation consisted of NR mixed with butadiene rubber, the TWP concentration decreased. Changing the type of CB also reduced the amount of TWP in the 2.5 µm size range. The TWP concentration in the specimens increased with increasing speed and vertical load. The TWP generated during the abrasion tests using wear testers and tire simulators exhibited similar trends. These findings suggest that modifying tire tread formulations can effectively control the distribution and amount of TWP generation.

## 1. Introduction

Particulate matter (PM) refers to inhalable dust that consists of particles with a diameter of ≤10 μm that float in the atmosphere [1]. PM is classified by size, with ≤10 μm-diameter PM being referred to as PM10 and ≤2.5 μm-diameter PM being classified as fine PM or PM2.5. PM can cause various diseases, such as conjunctivitis, keratitis, rhinitis, and bronchitis [2,3]. Although various sources of PM contribute to air pollution, exhaust emissions generated by internal combustion engines are a major source of air pollution [4]. In addition to exhaust emissions, non-exhaust emissions, such as those from brake and tire wear, are known to contribute to PM [5,6]. The utilization of particulate filters and the rise of new energy vehicles have contributed significantly to the minimization of vehicle exhaust particle emission [7,8]. Conversely, non-exhaust particle emissions have emerged as a pressing concern. Non-exhaust particles are progressively becoming the primary source of vehicular particle emissions, and their significance is escalating due to the ongoing decrease in exhaust emissions [9].

According to the 2008 National Emissions Inventory, the U.S. Environmental Protection Agency estimated that emissions from on-road vehicles accounted for 1% of the overall national average PM10, where tires were responsible for 15% of vehicle PM emissions [10]. Although there was no specific breakdown for tire wear, on-road vehicle emissions were estimated to contribute to 12% of PM10 emissions in the EU-27 countries in 2005 [11]. Recently, Euro 7, which aims to minimize these issues, added non-exhaust emission regulations to those related to exhaust emissions [12,13]. Although there are no clear evaluation methods or standards for tire PM, unlike brake PM, tire and rubber manufacturers are conducting various studies to address this issue. The new inclusions in Euro 7 regulations will likely involve emission standards for brakes with a limit of 7 mg/km for PM10 until 2034 and a limit of 3 mg/km from 2035 onwards. However, specific details regarding the evaluation method for tires, such as the concentration of PM, have not yet been disclosed. This is most likely due to the difficulty of analyzing the concentration of PM originating from tires [13].

Tire wear PM (TWP) originates from tire wear and tear during friction with the road surface which causes small particles to be released into the atmosphere. The mechanical wear of tire treads is known to occur primarily during the initial stages of the wear process at the interface between the tire and road, generating relatively large particles owing to the significant shear forces. Subsequently, as the shear forces stabilize, heat is generated at the interface between the tire and the road surface, and chemical wear of the tire occurs because of the generated heat. The morphology of TWP observed using scanning electron microscopy (SEM) included elongated, round, and irregular shapes. The EDS results showed that TWP had a broad-ranging elemental composition, including Al, Ba, C, Ca, Cl, Cr, Cu, Fe, and Zn, among other elements. [14,15]

Ha et al. conducted wear tests using different tire tread formulations to investigate tire wear and TWP according to the wear conditions. They found that a tread rubber consisting of 100% natural rubber (NR), which is commonly used for truck and bus tires, generates more PM than a synthetic rubber-blended tire tread, which is used to enhance abrasion resistance, because the synthetic rubber improved the stress distribution within the NR matrix at the μm scale [5].

Decreased wear of the NR and synthetic rubber blend can be explained in other ways. First, butadiene rubber (BR) has a lower glass transition temperature (−100 °C) than NRs. Therefore, BR rubber could have high resilience at an ambient temperature, causing less heat buildup under dynamic deformation. This property could indicate superior abrasion resistance [16]. Second, the coefficient of the friction of BR is smaller than that of NR. Therefore, BR is expected to have a smaller frictional force with the rough surface [17].

Lee et al. conducted wear tests using both actual roads and a laboratory simulator, capturing the particle materials generated by tires under braking conditions and analyzing particle size trends using SEM [18]. As TWP collected on the road contains several materials, such as road surface wear, dust, and vehicle exhaust, various shapes were collected, such as cubic, rounded, and irregular shapes of particles, and their origin was not distinguishable by the human eye.

Grigoratos et al. used a laboratory tire wear simulator to analyze the particle size distribution and chemical composition of the TWP generated by tire wear [19]. The PM10 concentrations consistently increased, followed by a quasi-stable level, while the PM2.5 concentration continuously increased without stabilizing. Different tire brands with the same tire wear rate (TWR) exhibited varying levels of wear and PM concentrations. Therefore, it was difficult to classify different brands based on the TWR.

Kreider et al. conducted both actual road and road simulator tests to distinguish between roadway particles, tire wear particles, and tread particles generated during wear and analyzed the composition and shape of the generated particles [20]. They reported that the morphologies of the TWP from on-road collection and tire simulations are quite similar. However, the TWP collected on the road are smaller on average. The chemical composition of TWP differed with the on-road generated particles because they contain various chemical compositions related to the source.

Capturing tire-derived PM on the road is difficult because of the high noise caused by various foreign substances, including exhaust gas, on the road surface. Therefore, analysis is usually performed using tire simulators. However, accurately replicating real road conditions is challenging, which can cause various difficulties [18,19,20].

As stated previously, several studies have used various approaches to analyze TWP, and the most common methods used involve ambient TWP measurements on open roads through trying tests and road simulator measurements. However, reliable standardized measurement methods are still limited [21,22].

This study aimed to examine the reproducibility of a previous study [5] and the pattern of PM emissions from truck and bus radial (TBR) tires by analyzing the TWP produced from various artificially worn tread specimens. Specifically, the effects of different blends of natural and synthetic rubber on tire treads as well as the particle size and distribution of carbon black (CB), which was used as a reinforcing agent, were compared. Additionally, the concentration distribution and characteristics of the PM generated through a specimen wear tester were measured. From these results, the selected tread formulation was determined for TBR and compared with reference TBR. These tires were analyzed using a tire wear simulator which simulates an environment similar to actual tire exposure while driving on pavement. To the best of our knowledge, this is the first research article that compares PM concentrations from different TBR tire-tread formulations using different wear devices.

## 2. Materials and Methods

### 2.1. Materials

The base rubbers used in this study were NR (SVR-10; dirt content of 0.1 wt%; Astlett Rubber Inc., Oakville, ON, Canada) and BR (NdBR-60; Mooney viscosity of 60; Kumho Petrochemical Co., Daejeon, Republic of Korea). CB was employed as the filler, specifically N234, OCI-1, and OCI-2. OCI-1 and OCI-B are pilot-produced CB that enhances dispersibility (with a target of N134, from OCI Company Ltd., Seoul, Republic of Korea). Table 1 summarizes the physical characteristics of the CB samples used in this study. While N234 is a common choice for truck and bus tires, this investigation aimed to determine whether narrow-aggregated size distribution (ASD) CB could reduce PM emissions from tires. To explore this hypothesis, OCI-1 and OCI-2 were utilized. ZnO and stearic acid (St/A; Sigma-Aldrich Corp., Seoul, Republic of Korea) were employed as activators, whereas N-(1,3-dimethylbutyl)-N′-phenyl-*p*-phenylenediamine (6PPD; Kumho Petrochemical Co., Daejeon, Republic of Korea) and 2,2,4-trimethyl-1,2-dihydroquinoline (TMQ; Sinopec Corp., Beijing, China) were used as antioxidants. The crosslinking agent was sulfur (Daejung Chemicals and Metals Co., Siheung, Republic of Korea) and the curing accelerator was N-tert-butyl-2-benzothiazylsulfenamide (TBBS; Shandong Yanggu Huantai Chemical Co., Ltd., Liaocheng, China). Additionally, a pre-vulcanization inhibitor, N-cyclohexylthiophthalimide (Shandong Yanggu Huantai Chemical Co., Ltd., Liaocheng, China), was applied.

### 2.2. Manufacture of Compounds and Vulcanizate

The compounds were prepared utilizing an internal mixer (300 cc, Mirae Scientific Instruments Inc., Gwangju, Republic of Korea) in accordance with the formulations and characteristics outlined in Table 2. The mixer was filled to 80% of its volumetric capacity. The input unit was expressed as parts per hundred rubber, and the quantity of each compound was proportionate to the amount of rubber introduced.

The cure characteristics of the compounds were evaluated based on the minimum torque (*T_min_*), maximum torque (*T_max_*), scorch time (*t*_10_), and optimal cure time (*t*_90_), which were measured using a moving die rheometer (MDR, Myung Ji Co., Seoul, Republic of Korea) for 30 min at 150 °C and a vibration angle of ±1°. Table 3 includes a summary of the blending process, in which the primary and secondary stages were initially heated to 100 and 60 °C, respectively, and the dumping temperatures for the primary and secondary stages were within the ranges of 150–155 °C and 80–90 °C, respectively. Once blending in each stage was complete, the materials were shaped into sheets using a two-roll mill.

The vulcanizates were prepared by pressing the compound in a hydraulic press at 150 °C for the optimal curing time (*t*_90_).

#### 2.2.1. Bound Rubber Content

Following the initial mixing phase, a portion of the compounds (0.2 ± 0.01 g) was deposited onto filter paper and submerged in 100 mL of toluene at 25 °C for 6 d to extract the unbound rubber. The toluene solution, which contained the extracted unbound rubber, underwent purification using acetone and subsequent drying. The content of the bound rubber was determined by comparing the weight of the sample before and after the experiment, according to the following calculation [23]:

(1) $$Bound\;rubber\;content\;\left(\%\right)=\frac{w_{fg}-w_{t}\left[\frac{m_{f}}{m_{f}+m_{r}}\right]}{w_{t}\left[\frac{m_{r}}{m_{f}+m_{r}}\right]}\times 100$$

where *w_fg_* is the filler and gel weight, *w_t_* is the specimen weight, *m_f_* is the weight fraction of the filler in the compounds, and *m_r_* is the weight fraction of the polymer in the compounds.

#### 2.2.2. Crosslink Density

To eliminate organic additives, present within the vulcanizate specimens, the specimens of dimensions 10 mm × 10 mm × 2 mm were successively submerged in tetrahydrofuran (99%, Samchun Chemical Co., Seoul, Republic of Korea) and *n*-hexane (95%, Samchun Chemical Co., Seoul, Republic of Korea) for a duration of 1 d each at 25 °C. The weight of the specimens was documented. Subsequently, the specimens were immersed in toluene at room temperature for 1 day, and the weight of the swollen specimens was measured. The total crosslink density was determined utilizing the Flory–Rehner equation, as expressed below [24]:

(2) $$\nu =\frac{1}{2M_{c}}=-\frac{lnln\;\left(1-V_{r}\right)+V_{r}+\chi V_{r}^{2}}{2\rho_{r}V_{s}\left(V_{r}^{\frac{1}{3}}-0.5V_{r}\right)}$$

where *ν* is the crosslink density (mol/g), *M_c_* is the average molecular weight between crosslink points (g/mol), *V_r_* is the volume fraction of rubber in the swollen gel at equilibrium, vs. is the molar volume of solvent (cm^3^/mol), *ρ_r_* is the density of the rubber sample (g/cm^3^), and *χ* is the polymer-solvent interaction parameter.

#### 2.2.3. Tan ẟ at 0 °C, 60 °C

The viscoelastic characteristics of the compounds were analyzed by determining the Tan ẟ values within the temperature range of −70 to 80 °C, employing a dynamic mechanical analyzer (DMA Q800, TA Instrument, New Castle, DE, USA) with a strain of 0.2% and a frequency of 10 Hz.

### 2.3. Preparation of Rubber Specimens and Tires

The specimens for PM measurement were prepared by curing the final masterbatch (mold size : 25 mm width × 25 mm length × 10 mm height) in a hydraulic press at 150 °C under the optimal curing time. The study aimed to investigate the effects of natural-to-synthetic rubber ratios and types of synthetic rubber on the generation of PM. A series of experiments were conducted to analyze the impact of these factors. OCI-1, OCI-2, and N234 were used to study the influence of the CB type on OCI-1 PM generation. The selected formula combination (K-0 and K-5), manufactured with the same composition, was produced by Hankook Tire and Technology as the TBR (size: 11R22.5) to prepare the specimen and was used for the simulator evaluation. Detailed information on the manufacturing process for TBR is not provided in this study because it is proprietary knowledge of the company.

### 2.4. Specimen Wear Device

**Figure 1:**
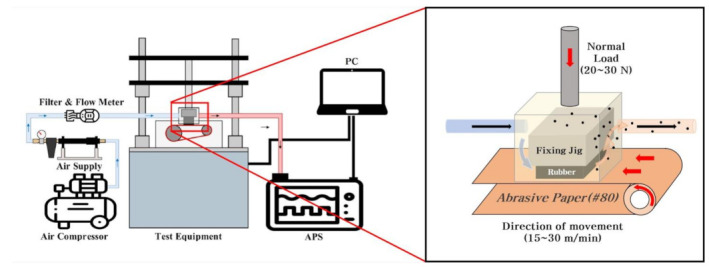
Schematic illustration of the rubber specimen wear tester and measurement equipment.

The wear tests were carried out on the specimens using a wear tester, as shown in Figure 1. The tester applied a vertical load of 20–30 N to the specimen which was then subjected to an 80-grit sandpaper belt with a mean grain diameter of 150–250 µm that rotated at a speed of 15–30 m/min, and each test was carried out for 5 min. The sandpaper belt (#80-grit) was used to simulate the roughness of an asphalt road following the SAE J1269 procedure [25,26]. The wear of the rubber in the chamber during testing is presented in Figure 1. It should be noted that among various tire driving modes, the tester was only capable of simulating the braking mode, where the specimen was fixed and the sandpaper belt rotated, thereby causing abrasion. These test conditions were harsher than those typically encountered on actual roads. The utilization of such testing apparatus enabled the measurement of TWP generation during the abrasion of rubber specimens in the laboratory. This setting provides a simple and convenient testing approach prior to tire tread development.

### 2.5. Tire Wear Simulator

An internal tire abrasion simulator (Daekyung Engineering, Ulsan, Republic of Korea) was used to evaluate the abrasion of the tire and generated PM while the tire rotated. This simulator consists of a rotating drum, closing chamber, and wheel-driving unit. The tire simulator using the ‘11R22.5’ tire can control the vertical load (100–−60,000 N), drum speed (0–150 km/h), camber angle (−10–10°), and slip angle (−5–5°). The inner diameter of the rotating drum is 3.8 m, and an interchangeable pavement is located along the inner surface of the drum. In particular, this simulator can change the pavement type between sandpaper, asphalt, and concrete. In this study, sandpaper (grit size: 80 mm) was used as the pavement. Abrasion tests were performed on three developed (K-5) and three control tires (K-0). Table 4 summarizes the test conditions.

### 2.6. Measurement of PM

**Figure 2:**
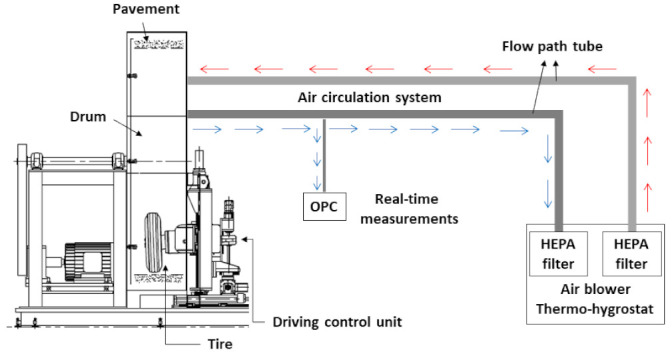
Schematic illustration of the tire wear simulator. OPC = optical particle counter.

The size distributions of the particles in the range from 0.5 to 20 µm in aerodynamic diameters was determined using an aerodynamic particle sizer (APS). PM can be classified into two categories, namely PM 2.5 and PM10, which represent particles with aerodynamic diameters <2.5 and 10 µm, respectively. Typically, PM is measured using two different methods: the gravimetric analysis of particles collected on a filter or substrate and real-time PM estimation using a light scattering method. The latter method was employed in this study due to its suitability for monitoring rapid fluctuations in PM levels. To prevent external air from interfering with the wear test outcomes, a clean and dry air supply, free of moisture or particulate impurities, was obtained by utilizing a regular air filter (TSI 3074 B, Shoreview, MN, USA) and an ultra-low penetration air filter (TSI 3140, Shoreview, MN, USA). The flow rate of air within the wear test chamber was maintained at a constant rate of 5 L/min and measured by a flow meter (TSI 5300, Shoreview, MN, USA). To minimize particle loss, a conduction tube was connected between the sampling port of the specimen wear device at the bottom of the chamber and the PM measuring equipment installed on it. An APS (TSI 3321, Shoreview, MN, USA) was used to measure the mass concentration, number concentration, and particle size distribution based on the wear of the rubber specimens. An optical particle counter (OPC; Grimm 11-D, Bayern, Germany) was used in the tire wear simulator to determine the PM concentration and its distribution at a sample flow rate of 1.2 L/min. Figure 2 shows a schematic of the installation. TWPs from the specimens were examined using SEM (Apreo S HiVac, Hillsboro, OR, USA) at 3–6 keV working voltage. To identify the composition of the particulates, an energy dispersive spectrometer (EDS, XFlash6l100, Pottsville, PA, USA) was used.

## 3. Results

### 3.1. Effect of Tread Prescription

**Figure 3:**
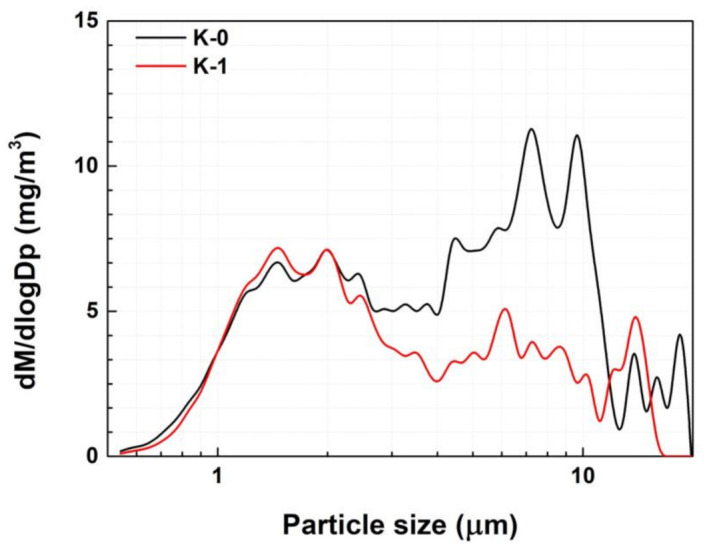
PM concentration distribution of K-0 and K-1.

**Figure 4:**
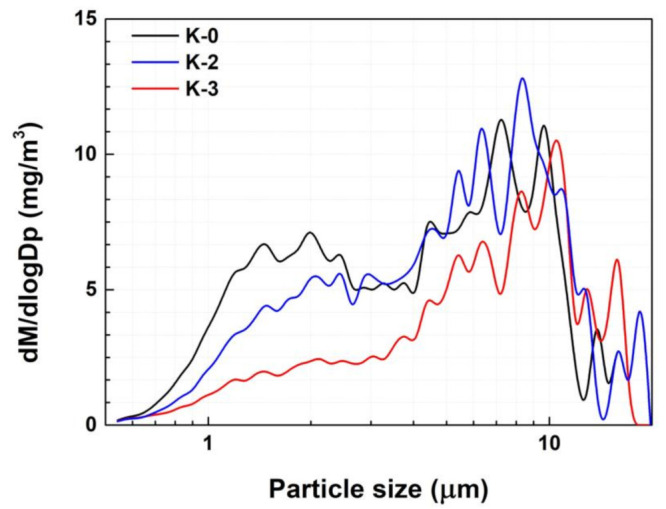
PM concentration of K-0, K-2, and K-3.

A previous study found that when NR 100% (K-0) was applied as the material for the tread of city bus tires, a large amount of PM 5–10 µm was generated [5]. However, it was observed that the PM concentration in that range decreased when it was mixed with BR (Figure 3). The study further explained that optimally sized BR particles can induce a multicraze initiation and termination mechanism capable of dissipating large impact energies. The NR containing a BR matrix exhibited more dispersed shear yielding which necessitated significant cooperative chain motion. Consequently, the incorporation of BR enhanced the stress distribution within the NR matrix [27]. The results also reveal that changing the type of CB could reduce the amount of PM in the 2.5 µm size range. The findings of previous computational simulations [5] indicate that an increased volume fraction in the rubber matrix leads to greater stress on the periphery of the matrix caused by CB. Therefore, with CB with a lower ASD and an unexposed surface area, rather than regular CB, the TBR tended to decrease (Figure 4) because large-sized CB particles can increase the stress gradient inside the rubber matrix and facilitate TWP generation. As mentioned in Section 2, OCI-1 and OCI-2 were less aggregated and had larger unexposed surface areas than N234. OCI-2, which had the lowest ASD, exhibited a reduced PM concentration of 2.0–3.0 µm.

**Figure 5:**
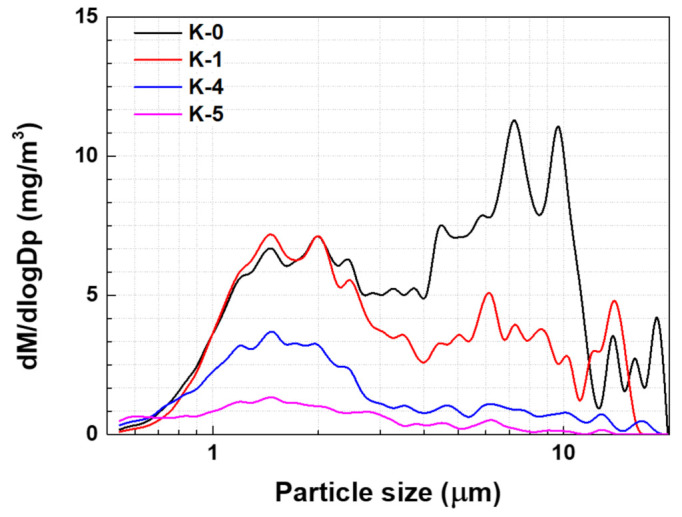
PM concentration of K-0, K-1, K-4, and K-5.

BR 100% (K-4) also exhibited a reduced PM concentration but was not as effective as the NR and BR alloy matrices (Figure 5). Based on the results, the explanations attributing the favorable wear characteristics of BR to its low *Tg* [16] and the reduction of TWP due to its low coefficient of friction [17] seem inadequate. Instead, it is appropriate to consider BR as an inhibiting crack propagation, similar to impact modifiers.

NR normally has inherent strength, large deformation capability, and resistance to crack propagation, whereas BR has better abrasion resistance, resilience, and resistance to crack initiation. Ghosh et al. reported that NR and BR alloy rubber has transition points that exhibit a better crack growth rate. Therefore, it is also possible that our test conditions were in this particular condition [28]. Future experiments are planned to further investigate the case and provide a comprehensive analysis of the related research.

### 3.2. Effect of Wear Conditions

**Figure 6:**
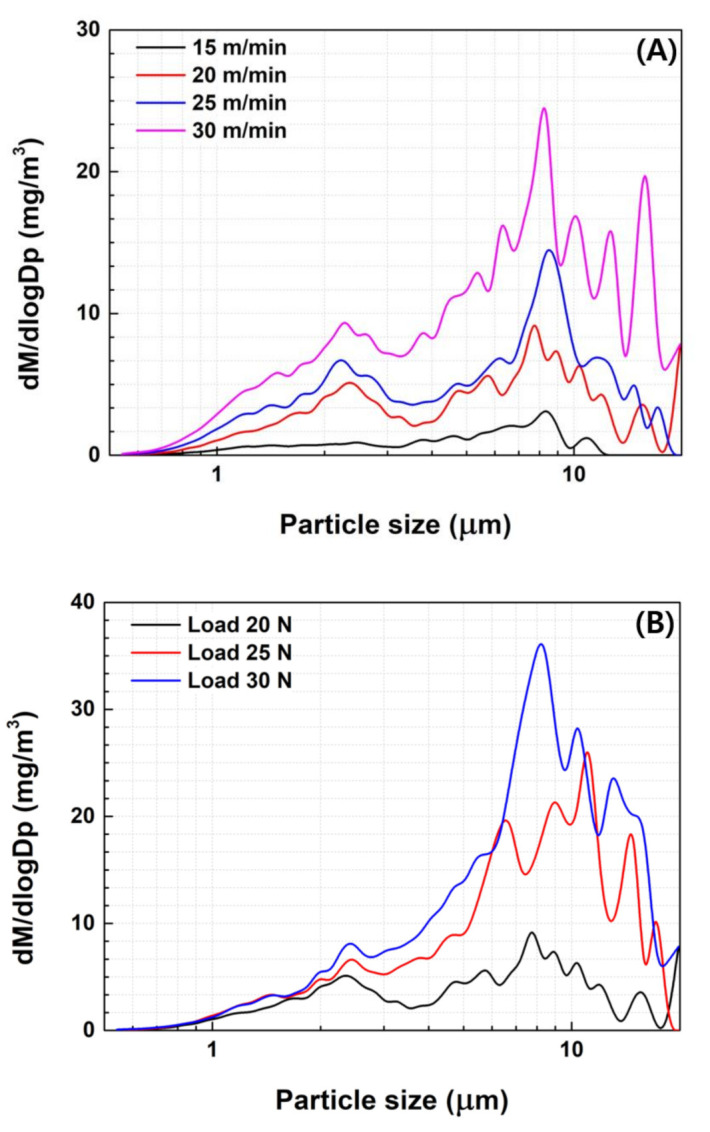
Average PM concentration with different (**A**) wear speeds and (**B**) loads.

Figure 6 compares the TWP concentrations generated from K-0 with varying wear rates and loads. It shows that an increase in the wear rate led to an increase in the TWP concentration under the same vertical load of 20 N. Under slow abrasion conditions (15 m/s), a relatively small amount of TWP was generated. The 2.0–3.0 µm PM concentration was very low, but the concentration of 8.0–10 µm PM was slightly higher. The concentration of 2.0–3.0 µm PM increased with an increasing abrasion speed.

Similarly, the specimen wear device was operated with vertical loads of 20, 25, and 30 N at a consistent speed of 20 m/s. The emitted TWPs were then measured to investigate the effect of the load on the TWP emission. The vertical load was linearly correlated to the PM concentration. A vertical load of 30 N resulted in a higher PM concentration throughout the detection area. These results showed a similar trend to that of a previous study [14].

Previous studies [18,29,30] reported that the tread wear rating (which is dependent on the tread prescription) did not affect the size distribution and exhibited a single-mode concentration distribution; however, the tires were all passenger car tires with BR-based mixtures and silica as a filler; therefore, the studied treads had a similar matrix rubber. Because many studies on TWP have focused on passenger car tires, the observation of a bimodal concentration distribution in NR-based commercial vehicle tires is unique. Moreover, when BR was added, a unimodal concentration distribution was observed, demonstrating that the prescription of tire treads can control the distribution of TWP.

### 3.3. Tire Wear Simulator Results

**Figure 7:**
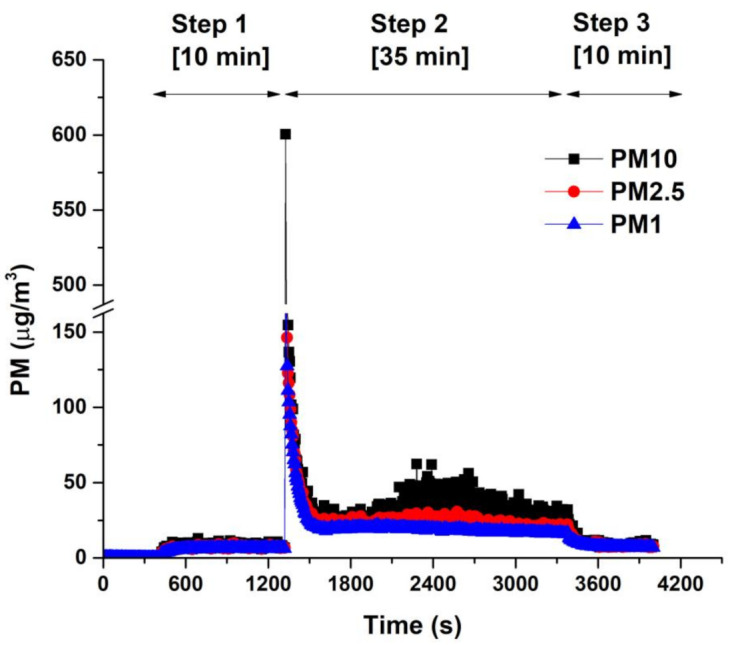
PM generated from the tire simulator over time.

Although clear results were obtained from the above experiment, the experimental outcomes have limitations regarding accurately depicting tire wear during vehicle operation on roads. This is because the specimen wear tester, which abrades rubber specimens by moving the sandpaper belt at the bottom while the sample is fixed at the top, results in very severe tire slip conditions. Therefore, a tire wear simulator (Figure 2) was used to minimize the problems that occurred in the previous experiment. Figure 7 shows the overall concentration of the PM generated during the entire experimental period. When the simulator drum was operated, the PM concentration increased slightly, and when the tires made contact with the road surface, the PM concentration increased significantly. After stabilization, PM continued to be generated as the tires contacted the road surface. The increased concentration of PM at the beginning of equipment operation is believed to be due to the dust generated by the movement of the equipment. The rapid increase in PM concentration after the tire contacted the road surface is presumed to be caused by the strong torque applied to the tire during initial contact with the road surface. After the initial tire –road contact, the PM concentration decreased significantly and exhibited a consistent and relatively low PM concentration until the experiment was stopped.

**Figure 8:**
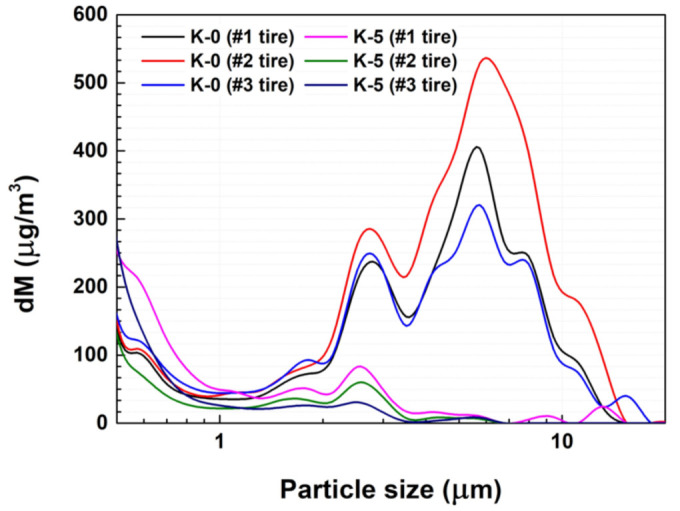
PM concentration distribution of K-0 and K-5 from the tire wear simulator.

The baseline for the PM concentration in this experiment was set as the amount generated by equipment operation before the tire made contact with the road surface. The PM concentration that increased excessively when the road surface was first contacted was excluded to compare the TWP concentration generated during travel of the tire. The selected tread formulations, K-0 (reference) and K-5 (lowest TWP), were used to make a tire for simulations. The PM2.5/PM10 ratio indicates the ratio between the relatively small PM2.5 and the relatively large PM10 in the measured PM content. The experimental results (Table 5 and Figure 8) indicate that the PM2.5/PM10 ratio for K-0 (a tread with NR 100% application) was lower due to the relatively high PM10 concentration. However, the ratio was higher for K-5 (a specimen with a tread containing a mixture of NR and BR), mostly because the PM10 concentration decreased. K-0 exhibited a larger amount of wear than K-5. As TWP can be generated when rubber undergoes wear, specimens with more wear also tend to have higher concentrations of TWP [15]. This trend was also observed in the simulator results.

As previously mentioned, the concentration of PM generated by the tire when the rotating drum moved against a stationary specimen was analyzed using the wear tester. However, there are limitations to accurately describing tire wear on actual roads. Therefore, an internal tire simulator was constructed, and experiments were conducted to compare and verify the concentration of PM generated when the tire rotated and abraded against the road. Figure 8 shows the distribution of the PM concentration generated when the tire was driven in the tire simulator. In the case of TBR tires, when the tire wear experiment was conducted using K-0 (NR 100%), the concentration gradient of the TWP was bimodal, and it exhibited relatively high concentration values of 2–3 and 5–8 µm. The PM2.5/PM10 ratio of K-0 was low because the PM10 concentration was high. In contrast, for K-5, the concentration gradient of the TWP was unimodal. This concentration distribution was very similar to that of the specimen wear device. The reduction in TWP can also be explained by the combination of the lower *Tg,* the low friction coefficient of BR, and the cracking inhibition properties of BR in the NR matrix.

### 3.4. Morphological Comparison of TWP

**Figure 9:**
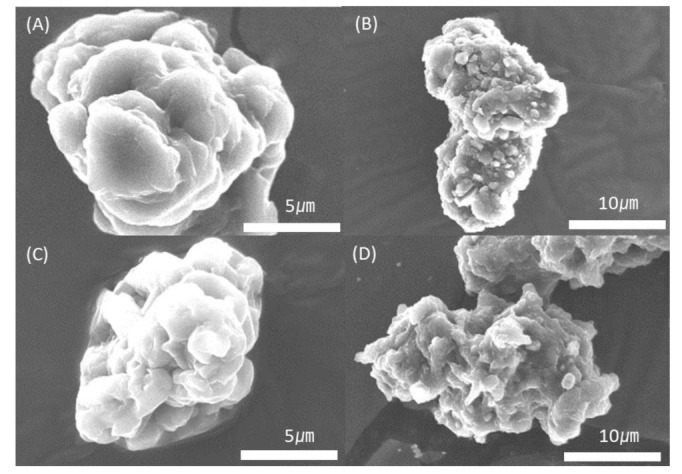
Scanning electron microscopy images of the TWP from K-0 (**A**,**B**) and K-5 (**C**,**D**).

**Figure 10:**
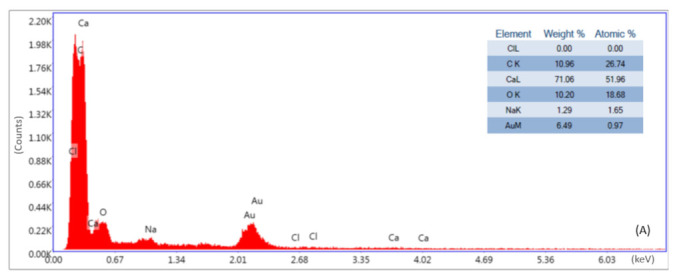

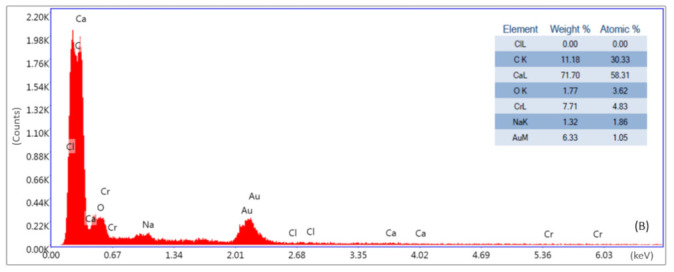
EDS results of the TWP from K-0 (**A**) and K-5 (**B**).

The TWP generated by the wear-testing machine and tire wear simulator was analyzed using SEM (Figure 9) and EDS (Figure 10). The TWP exhibited elongated, round, and irregular shapes. However, there was no discernible variation according to the prescription, and it seems unlikely that any specific conclusions can be drawn from the worn-out specimens. However, previous studies have reported that TWP resulting from mechanical wear exhibits a coarse particle shape, whereas TWP formed through the gas-to-particle conversion process exhibits relatively small particles with a round/irregular morphology [20]. TWP had various elemental compositions, including elements such as C, Ca, Cl, Cr, Mn, Zn, and Na (Figure 10 shows selected EDS images, and the Au peak in the pattern is due to the Au coating on the specimen), which were mostly consistent with previous research results. A previous study identified the composition of C-Si by analyzing passenger car tires [18]. However, in this experiment, the used tire tread contained CB as the sole reinforcement; therefore, Si was not detected, and C was the dominant element in the TWP.

## 4. Conclusions

In this study, the TWP from tread rubber with different formulations was analyzed, and the concentration of TWP generated using different wear devices was compared. This study confirmed the following:

- The TWP generated by tire wear had different concentration distributions depending on the composition of the tire tread.
- The patterns of the TWP artificially generated using the wear-testing device and simulator were very similar.
- When using both types of testing equipment, a bimodal TWP concentration distribution was observed in the NR-only matrix, whereas the PM concentration above PM5.0 decreased and exhibited a unimodal concentration distribution for the alloy of NR and BR.
- Both the TWP concentration and amount of wear decreased for the tire tread mixed with NR and BR.
- The amount of generated PM tends to increase as the tire wear increases.

These findings suggest that modifying tire treads can effectively control the distribution of TWP. Additionally, we plan to obtain data that predict tire-generated PM in a sample state and incorporate it into our comparison of PM generation patterns. This approach will allow us to evaluate the accuracy of the tire simulator in predicting PM emissions under various driving conditions.

## Figures and Tables

**Table 1:** Properties of the CB samples.

Property		N234	OCI-1	OCI-2
IA (iodine adsorption)	mg/g	119	143	139
N_2_SA (nitrogen surface area)	m^2^/g	115	138	144
OAN (oil absorption number)	cc/100 g	125	118	131
Tint (ITRB *)	%	123	136	129

* Industry tint reference black.

**Table 2:** Composition of the tread compounds (parts per hundred rubber) and properties.

Material	K-0 (Ref)	K-1	K-2	K-3	K-4	K-5
NR ^a^	100	70	100	100	-	70
NdBR60	-	30	-	-	100	30
CB (N234)	55	55	-	-	55	-
CB (OCI-1)	-	-	55	-	-	-
CB (OCI-2)	-	-	-	55	-	55
Common formulations	TDAE oil ^b^ (5), ZnO (4), St/A (3), 6PPD (2), TMQ (1), Sulfur (1.3), TBBS (1), PVI (0.3)					
Bound rubber contents (%)	34.5	35.6	37.1	39.2	14.6	36.9
Crosslink density (10^−5^ mol/g)	8.14	8.71	8.08	8.28	9.01	9.61
DIN abrasion loss (mg)	91.3	43.8	88.3	86.7	5.8	34.5
Tan δ at 0 °C	0.181	0.161	0.168	0.170	0.121	0.155
Tan δ at 60 °C	0.122	0.109	0.116	0.113	0.114	0.107

^a^ Standard Vietnamese Natural Rubber SVR-10, dirt content of 0.1 wt%. ^b^ Treated Distillate Aromatic Extracted. *Abbreviations*: 6PPD, N-(1,3-dimethylbutyl)-N′-phenyl-p-phenylenediamine; CB, carbon black; DIN, Deutsche institut fur Normung; NR, natural rubber; PVI, pre-vulcanization inhibitor; St/A, stearic acid; TBBS, N-tert-Butyl-2-benzothiazylsulfenamide; TDAE, treated distilled aromatic extract; TMQ, 2,2,4-trimethyl-1,2-dihydroquinoline.

**Table 3:** Mixing procedure used for manufacturing the compounds and vulcanizates.

Step	Time (min:s)	Action
First Stage	0:00–1:30	NR or BR mastication (initial temperature: 100 °C)
	1:30–2:30	Add 50 wt% of CB and 50 wt% of oil
	2:30–3:30	Remaining CB and oil were added.
	3:30	Add St/A, ZnO, 6PPD, and TMQ
	3:30–5:30	Extra mixing and dump (dump temperature: 150–155 °C)
Second Stage	0:00–0:30	Master batch from first stage (initial temperature: 60 °C)
	0:30–2:30	Curatives and dump (dump temperature: 80–90 °C)

*Abbreviations*: 6PPD, N-(1,3-dimethylbutyl)-N′-phenyl-p-phenylenediamine; BR, butadiene rubber; CB, carbon black; NR, natural rubber; St/A, stearic acid; TMQ, 2,2,4-trimethyl-1,2-dihydroquinoline.

**Table 4:** Tire wear simulator test conditions.

Test Stage	Driving Conditions
Step 1. 60 km/h tire rotation without pavement contact, 15 min Step 2. 60 km/h tire rotation with pavement contact, 35 min Step 3. 60 km/h tire rotation without pavement contact, 10 min	Speed: 60 km/h Vertical load: 4900 N Slip angle 1.5°

**Table 5:** PM concentrations of K-0 and K-5 and the amount of wear.

Size	K-0 1st Run	K-0 2nd Run	K-0 3rd Run	Ave	K-5 1st Run	K-5 2nd Run	K-5 3rd Run	Ave
PM10 (μg/m^3^)	77.7	72.5	68.5	72.9	26.4	30.4	34	30.3
PM2.5 (μg/m^3^)	42.1	44	40.8	42.3	21.6	24.3	25.7	23.8
PM2.5/PM10	0.54	0.61	0.60	0.58	0.82	0.80	0.76	0.79
Amount of wear (kg)	0.22	0.2	0.2	0.21	0.14	0.16	0.16	0.15

## Data Availability

The data presented in this study are available upon request from the corresponding author.
